# Beneficial effects of empagliflozin on hematocrit levels in a patient with severe anemia

**DOI:** 10.1007/s40199-021-00417-5

**Published:** 2021-09-21

**Authors:** Jan Budzianowski, Janusz Rzeźniczak, Jarosław Hiczkiewicz, Dominika Kasprzak, Anna Winnicka-Zielińska, Bogdan Musielak, Konrad Pieszko, Paweł Burchardt

**Affiliations:** 1 Collegium Medicum, University of Zielona Góra, Ul. Zyty 28, 65-046 Zielona Góra, Poland; 2 Clinical Department of Cardiology, Nowa Sól Hospital, Nowa Sól, Poland; 3 Department of Cardiology, J. Struś Hospital, Poznań, Poland; 4 Department of Hypertensiology, Angiology and Internal Medicine, Poznań University of Medical Sciences, Poznań, Poland

**Keywords:** Empagliflozin, Hematocrit, Anemia

## Abstract

**Introduction:**

Sodium-glucose cotransporter (SGLT2) inhibitors may additionally benefit patients with diabetes by improving their erythropoiesis followed by the elevation of hemoglobin and hematocrit levels.

**Reason for the report:**

In the case described, severe normocytic normochromic anemia was resolved when empagliflozin had been introduced to the therapy.

**Case summary:**

A 78-year-old male patient was admitted to our hospital with a non-ST-segment elevation myocardial infarction. His past medical history included diabetes, right coronary artery angioplasty, myocardial infarction and paroxysmal atrial fibrillation which required anticoagulant treatment. When examined, severe normocytic normochromic anemia was also diagnosed. About two years prior to his admission, the patient began suffering from persistent anemia despite the modification of his anticoagulant therapy with warfarin, rivaroxaban and dabigatran. An extensive evaluation failed to provide an explanation for his anemia.

**Outcome:**

Eventually, only the introduction of empagliflozin successfully increased the values of hemoglobin and hematocrit. Therefore, it transpires that SGLT2 enhances erythropoietin (EPO) secretion which subsequently raises hematocrit levels in patients with severe anemia.

**Graphic abstract:**

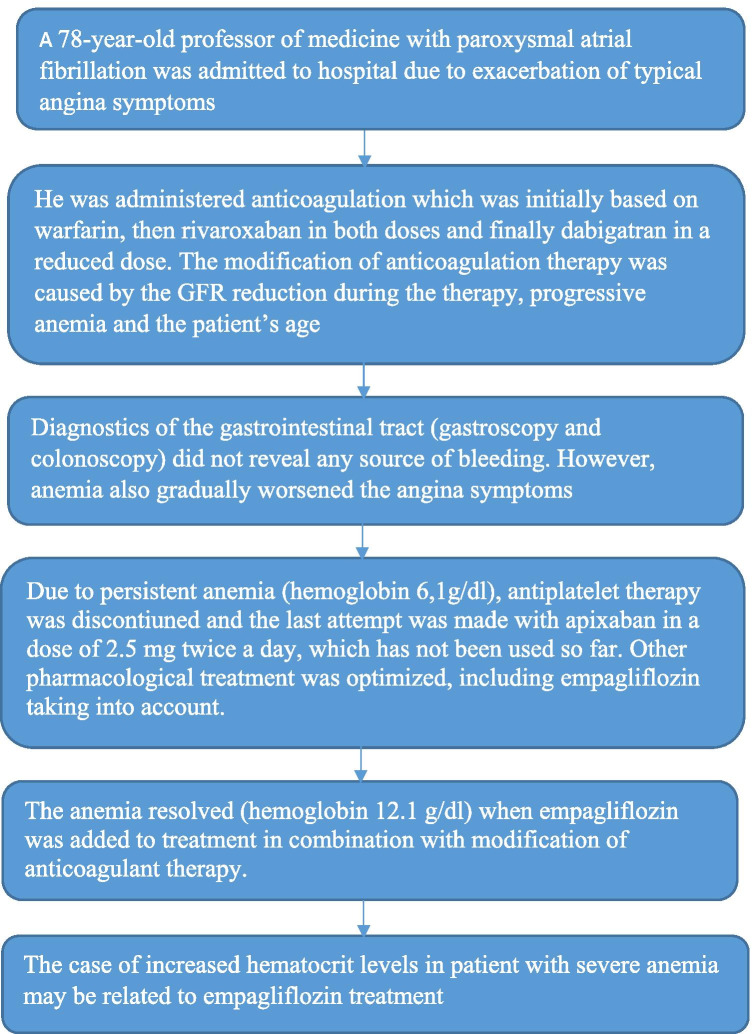

## Introduction

SGLT2 (sodium-glucose cotransporter) inhibitors as a class of drugs have made an accidental, but extremely significant breakthrough in cardiology. Their multidirectional pleiotropic benefits on the cardiovascular system translate into a reduced incidence of total mortality, heart failure hospitalization and nephroprotection [1]. The results of the studies showing that these drugs also cause an increase in hematocrit levels have turned out to be extremely interesting [2]. It has been suggested that the mechanism of this effect may be secondary to the general diuretic properties (natriuresis, glucosuria) of SGLT2 inhibitors.

## Reason for report

A growing body of data indicates that SGLT2 inhibitors may increase hematocrit by an enhanced synthesis of erythropoietin (EPO), which ultimately promotes myeloid erythropoiesis [2, 3]. This case reveals the remarkable effect of empagliflozin used for diabetics, which has additional properties of enhancing erythropoiesis and raising hematocrit.

## Case presentation

We observed for the first time such an effect in a 78-year-old man, a professor of medicine, who was admitted to hospital due to an exacerbation of typical angina symptoms and non-ST-segment elevation myocardial infarction (NSTEMI). In 2013 the patient had a history of right coronary artery (RCA) angioplasty. He subsequently required a pacemaker implantation due to a 3rd degree atrioventricular block. Moreover, he suffered from diabetes, amiodarone-induced hypothyroidism, paroxysmal atrial fibrillation and required an obligatory anticoagulant treatment according to the CHA_2_DS_2_-VASC score. His anemia was diagnosed for the first time 2 years earlier when the patient had type 2 NSTEMI. His anticoagulation was initially based on warfarin, then rivaroxaban (in both doses) and finally dabigatran (in a reduced dose). The modification of anticoagulation therapy was based on the reduction in the glomerular filtration rate (GFR) (on admission the creatinine and GFR level was 174 µmol/L and 33 mL/min respectively according to the Cockcroft–Gault formula), the patient’s progressive anemia and age [4]. The diagnostics of gastrointestinal tract (gastroscopy and colonoscopy) did not reveal any source of bleeding. However, his anemia also gradually exacerbated angina symptoms.

Two years earlier and during his later hospitalization, due to additionally detected high concentrations of ultra-sensitive troponin, coronary angiography for the management of acute coronary syndromes in patients without a persistent ST-segment elevation was performed in accordance with the ESC guidelines [5]. Coronary angiography revealed an occlusion in the left circumflex artery and border left anterior descending artery stenosis, which was hemodynamically insignificant. Echocardiography showed no segmental contractility disorders during the entire observation period. However, a left ventricular ejection fraction was estimated at about 45%.

Due to anemia, the decision was made to discontinue the antiplatelet therapy with aspirin and clopidogrel and the last attempt was made with apixaban introduced in a dose of 2.5 mg twice a day (weight 81 kg, creatinine level 174 µmol/L), which has not been used so far.

Other pharmacological treatment was optimized, including: Levothyroxine tablet—75 µg once daily, Pantoprazole tablet—20 mg once daily, Torasemide tablet—2.5 mg once daily, Bisprolol tablet—2.5 mg once daily, Telmisartan tablet—40 mg once daily, Eplerenone tablet—25 mg once daily, Rosuvastatin tablet—20 mg once daily, Trimetazidine tablet—35 mg twice daily, Isosorbide Mononitrate prolonged release tablet—25 mg once daily. Moreover, a 10 mg empagliflozin tablet (taken once a day and manufactured by Boehringer Ingelheim International GmbH, Ingelheim, Germany) was added to the therapy taking into account the beneficial effects of the drug on the cardiovascular system.

**Fig. 1:**
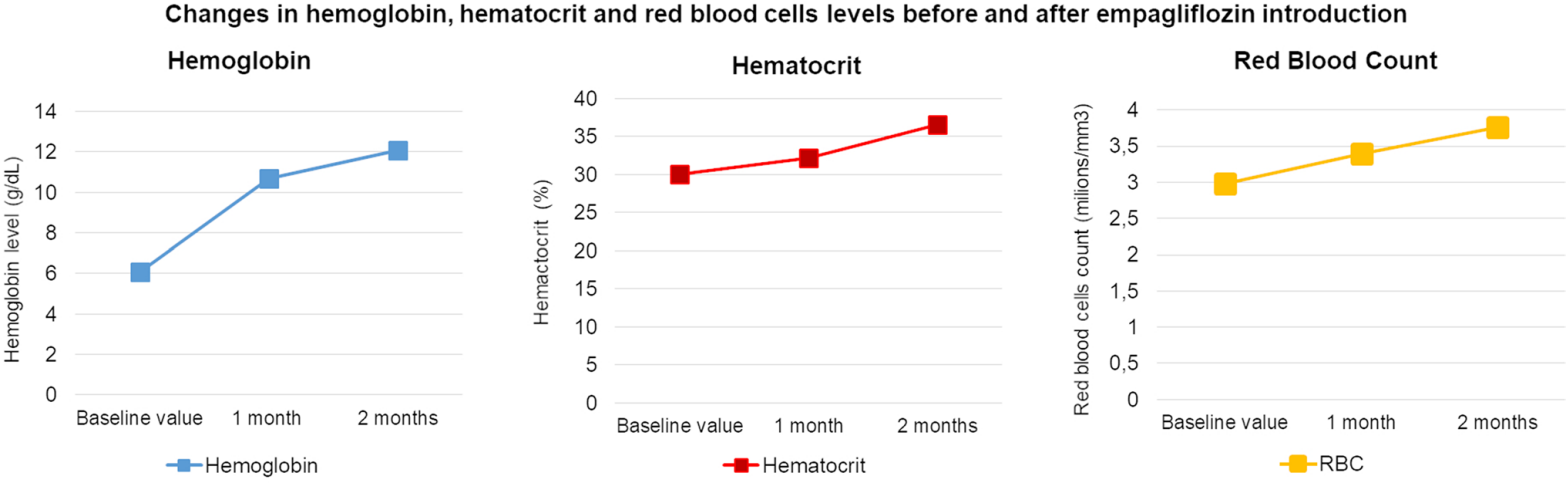
Changes in hemoglobin, hemocrit and red blood cells levels before and after empagliflozin introduction

Moreover, the patient received two units of packed red blood cells (PRBCs) based on the last complete blood count (CBC) obtained during his admission: HGB 6.1 g/dL (13.5–18 g/dL), RBC 2.98 × 10^12^/L (3.9–5.7 × 10^12^/L), HCT 30.03% (38.0%–55.0%), MCV 93fL (80–103 fL), MCH 32.4 pg [27.0–34.0 pg], MCHC 34.8 g/dL (31.0–37.0 g/dL), RDW 15.9% (11%–16%), Fe 12.2 µmol/L (10.7–28.3 µmol/L), vitamin B_12_ 348 pg/mL (187–883 pg/mL), Folic acid 5.6 ng/mL (3.1–20.5 ng/mL). Additionally, regular laboratory tests were recommended. The next CBC was performed one month after leaving hospital: RBC 3.4 × 10^12^/L (3.9–5.7 × 10^12^/L), HGB 10.7 g/dL (13.5–18 g/dL), HCT 32.2% (38.0%–55.0%) and after two months: RBC 3.76 × 10^12^/L (3.9–5.7 × 10^12^/L), HGB 12.1 g/dL (13.5–18 g/dL), HCT 36.6% (38.0%–55.0%) (Fig. 1).

For over the two-year follow-up period, anemia persisted despite the modification in the anticoagulant therapy. Also, before his admission an oral iron therapy with 320 mg of ferrous sulfate taken once a day was prescribed. However, due to side effects, the treatment was discontinued after 4 months. The erythropoietic effect was only identified after empagliflozin had been introduced.

## Discussion

A 78-year-old man who had coronary artery disease, diabetes, paroxysmal atrial fibrillation was referred to our hospital due to NSTEMI. He had multiple comorbidities and had a baseline hemoglobin concentration of approximately 6.1 g/dL despite the modification of anticoagulant therapy. About 2 years prior to his admission, the patient began having recurrent episodes of severe anemia and endoscopic assessment revealed no overt signs or symptoms of bleeding. Based on the clinical and lab data, normocytic normochromic anemia was diagnosed. It subsided only through the introduction of empagliflozin into his diabetes treatment.

Empaglifozin is a SGLT2 inhibitor which acts by inhibiting the glucose reabsorption in the proximal tubule of the kidney. A breakthrough results from the EMPA-REG OUTCOME (Empagliflozin Cardiovascular Outcomes Event Trial in Type 2 Diabetes Mellitus Patients—Removing Excess Glucose) revealed that patients with type 2 diabetes at high risk of cardiovascular events had a significantly early reduction in major cardiovascular and renal outcomes [6]. Moreover, a subanalysis of EMPA-REG OUTCOME Trial suggested that an elevation of hematocrit during the empagliflozin therapy had the strongest association with the reduction of cardiovascular death [7]. Sano et al. proved that an increase in hematocrit during the SGLT2 inhibitors therapy may be associated with the recovery of tubulointestinal function and an increased production of EPO by “neural crest-derived” fibroblasts [3]. EPO, however, is expected to affect the system much more widely, through a mechanism typical for other cytokines and may act favorably on the cardiomyocyte mitochondrial function, angiogenesis, inflammation and myocardial tissue oxygen delivery [8]. One of the limitation of our study was the fact that we did not check the EPO levels before and during the treatment. However, in this case, the elevated hematocrit levels may be associated not only with the reduction in the intensity of anticoagulant therapy, but also with the addition of empagliflozin to the treatment.

## Outcome

In the case of this 78-year-old man, anemia subsided when empagliflozin was added to the treatment in combination with the modification of anticoagulant therapy.

## Conclusion

Increased hematocrit levels in patients with severe anemia may be related to the empagliflozin treatment. To our knowledge, this is the first case report indicating the beneficial erythropoietic effect of empagliflozin leading to an increase in hematocrit levels.

## Abbreviations

EPO: Erythropoietin
GFR: Glomerular filtration rate
HCT: Hematocrit
HGB: Hemoglobin
MCHC: Mean corpuscular hemoglobin concentration
MCV: Mean corpuscular volume
NSTEMI: Non-ST-segment elevation myocardial infarction
PRBCs: Packed red blood cells
RBC: Red blood cell
RDW: Red cell distribution width
SGLT2: Sodium-glucose cotransporter
